# Analysis of Risk Factors for in‐hospital Death in Elderly Patients with TEXAS Stage 3 and 4 Diabetic Foot Ulcers after Tibial Transverse Translation: A Case–Control Study

**DOI:** 10.1111/os.13908

**Published:** 2023-10-10

**Authors:** Xiaofang Ding, Yusong Yuan, Hailin Xu, Zhengwei Jing, Hao Lu, Yuanli Wang, Junlin Zhou

**Affiliations:** ^1^ Department of Orthopaedics, Beijing Chaoyang Hospital Capital Medical University Beijing China; ^2^ Beijing Longfu Hospital Beijing China; ^3^ Department of Orthopaedic Surgery China‐Japan Friendship Hospital Beijing China; ^4^ Department of Trauma and Orthopaedics, Peking University People's Hospital Peking University Beijing China; ^5^ National Center for Trauma medicine Beijing China; ^6^ Diabetic Foot Treatment Centre, Peking University People's Hospital Peking University Beijing China; ^7^ Department of Health Policy and Management, School of Public Health Peking University Beijing China

**Keywords:** Case Control Study, Death, Diabetic Foot, In Hospital, Tibial transverse transport

## Abstract

**Objective:**

Chinese physicians developed the Tibial Transverse Transport (TTT) technique to treat diabetic foot ulcers with more than 90% effective rate. But this method still could not avoid the in‐hospital death of patients. This study adopted a case‐control study to explore the risk factors of in‐hospital death in elderly patients with chronic ischemic diabetic foot after receiving TTT treatment.

**Methods:**

A total of 54 patients were included in the study from January 1, 2017 to April 30, 2021, by being paired with the cases in case group with their demographic data and results of blood routine, liver and kidney function. There were nine patients in case group with six male and three male. Forty‐five patients were selected in control group according to gender and diabetes type with 30 male and 15 female. Single factor logics regression analysis was used to explore the risk factors and odd ratios (OR) of in‐hospital death in patients. The nomogram and decision curve analysis (DCA) had been done by R Studio software.

**Results:**

The study found that age, course of diabetic foot, small dense low‐density Lipoprotein (smLDL), homocysteine (Hcy), superoxide dismutase (SOD), and prealbumin (PA) were risk factors for in‐hospital death of patients. The smLDL had the highest risk. The nomogram showed that PA accounted for the largest proportion in the death risk factors. The results of DCA proved that above six risk factors were the risk factors for patients with TEXAS Stage 3 and 4 diabetic foot ulcers.

**Conclusion:**

In the future diagnosis and TTT treatment for diabetic foot ulcers, doctors need to pay close attention to age, course of diabetic foot, smLDL, Hcy, SOD, and PA.

## Graphical abstract

Chinese physicians developed the Tibial Transverse Transport (TTT) technique to treat diabetic foot ulcers with more than 90% effective rate. But this method still could not avoid the in‐hospital death of the patients. The study found that age, course of diabetic foot, small dense low‐density Lipoprotein (smLDL), homocysteine (Hcy), superoxide dismutase (SOD), and prealbumin (PA) were risk factors for in‐hospital death of patients.

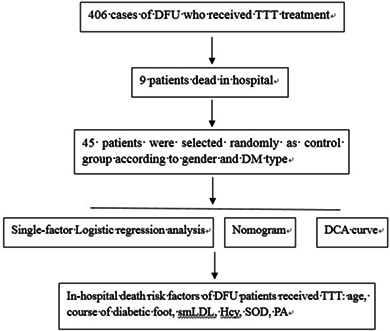

## Introduction

Diabetic foot (DF) is a common complication of diabetes, and about 15% of diabetic patients would suffer from DF infection or gangrene.^1^ The global prevalence rate of DFU was 6.3%, and the prevalence rate of diabetic foot ulcer (DFU) in some regions was as high as 16.6%.^2^ There were more males than females, and more type 2 diabetes than type 1 diabetes.^2^, ^3^ The cost of DF treatment is huge. The cost of treatment for DF in the United States accounted for one‐third of the cost of treatment for diabetes mellitus patients,^4^ and even exceeded the cost of some cancer treatments.^5^

There is currently no specific treatment for chronic ischemic DF ulcers. The guidelines for DF recommend a lot of treatment methods. However, chronic ischemic DF ulcers still need further breakthroughs.^6^, ^7^ Tibial transverse transport (TTT) is a surgical method developed by Chinese orthopedists based on Ilizarov's stress‐tension law to treat chronic ischemic DF ulcers. Chinese physicians applied periodic lateral transport and reduction of bone flaps^8^ to the treatment of lower limb ischemic ulcers, and named this method TTT. This procedure has been widely used in China. It has a good therapeutic effect on ischemic lower extremity ulcers.^9^, ^10^

TTT technology has been proved to be effective in promoting the healing of severe DF ulcers in a number of retrospective studies. At the same time, it is believed to promote limb angiogenesis and improve limb microcirculation.^11^ Even so, death is still an important adverse outcome for patients with severe DFU.^12^ How to avoid this outcome has become a key issue for TTT to be widely used in clinical practice.

Our team has used TTT technique to treat 406 cases of chronic DFU. There were nine cases of in‐hospital deaths during the early application stage of this technique. This study took a case–control study to: (i) explore the risk factors that lead to in‐hospital death of elderly patients; and (ii) provide a reference for the follow‐up treatment of other similar patients.

## Methods

### Patients

Patients were included in the study if they met the following requirements: (i) judged according to the criteria for DF as specified in the DF Prevention and Control Guidelines published in China in 2019, the patient meets the requirements in the guidelines; (ii) indications for TTT surgery were met and contraindications to surgery were excluded; (iii) patients who underwent TTT surgery; and (iv) having complete clinical information.

A total of 406 patients with DFU were treated at Peking University People's Hospital and Beijing Longfu Hospital in Beijing who received TTT treatment from January 1, 2017 to April 30, 2021 according to the including incision method mentioned above, of which nine patients died in‐hospital. The control group was drawn at a ratio of 1:5 among the remaining patients who received the same treatment but did not die in the hospital. The criteria for extracting the control group were the same gender and diabetes type. A total of 45 control groups were obtained. This study has been approved by Longfu Hospital Ethic Committee with the number of LFYYLL‐2021‐24.

### Treatments

All patients underwent surgical treatment of TTT. Antibiotics were routinely used to control infections after surgery^13^ and the internal medicine department continues comprehensive treatment to control blood glucose and treat original comorbidities.

The specific surgical steps are as follows^14^ Following nerve block anesthesia, the patient was placed in the supine position, and the affected limb was routinely disinfected. In the anteromedial area of the proximal tibia of the affected limb, the external fixation frame was compared with the area along the midline of the medial longitudinal axis of the proximal tibia. Subsequently, two 3.0 Steinmann pins were inserted through the single layer of cortical bone. The skin was cut along the long axis with the 3.0 Steinmann pin as the center, and the subcutaneous tissue was separated bluntly to expose the periosteum. The Steinmann pins were used as the center point for the drilling on four sides with a 2.0 drill bit and use of a rapid osteotomy device; the length of each side was 2.5 cm. Subperiosteal osteotomy was performed with a 5 mm narrow bone knife at an angle of 15°–30° to the bone surface. Attention was paid to protect the blood supply of the periosteum during this procedure. The external fixators were fixed with 4.0 Steinmann pins at the distal and proximal ends. The subcutaneous tissue and skin were sutured.

### Factors and Definition

Preoperative laboratory test results of blood routine, liver and kidney function, current illness history, and life history were collected. Cerebrovascular disease: Diagnosed by brain MRI or CT. Hypertension: The systolic blood pressure is greater than 120 mmHg or the diastolic blood pressure is greater than 80 mmHg. The following conditions were checked for. Carotid atherosclerosis: detection of plaques in patients by B‐Mode ultrasonography. Fundus atherosclerosis: color doppler ultrasound of fundus arteries confirmed that there were hardened plaques. Renal insufficiency: serum creatinine is greater than 133 μmol/L.

### Statistical Analysis

Statistical analysis was performed on the research data using SPSS 20.0 (IBM Armonk, NY, USA) statistical software. Measurement data were expressed as mean ± standard deviation (SD), and OR with 95% confidence interval (CI) was calculated. Single‐factor logistic regression analysis of clinical data was performed. *p* < 0.05 was considered statistically significant.

The data set was oversampled using the Synthetic Minority Over‐sampling Technique (SMOTE), and then randomly divided into a training set (75%) and a validation set (25%). A histogram was constructed using the training set, and the performance of the histogram was validated using the validation set. Multifactor regression analysis was employed to select variables from the data set, and variables with *p* < 0.05 were included in the model. The column line chart prediction model and clinical prediction curve were established using the “rms” and “ggDCA” packages in the R‐Studio software (Version 3.6.2, R Foundation for Statistics Computing, Vienna, Austria). The model's consistency was determined by constructing a calibration plot using 1000‐fold cross‐validation. The predictive ability of the column line chart was evaluated using 10‐fold cross‐validation. The clinical utility of the model was evaluated using decision curve analysis. The same methods were used to further validate the model's ability in the test set.

## Results

### In‐hospital Death Risk Factors

A total of 54 patients were enrolled in this study. The epidemiological history of the patients showed that the older the age, the higher the risk of death. For every 1 year increase in age, the risk of death increased by 1.351 times. The course of DF is also a risk factor. The longer the course of the disease, the higher the risk of death. For every additional month of DF, the risk of death increases by 1.968 times (Table 1).

**TABLE 1:** Sociodemographic details and diabetes characteristics of patients

Characteristic	Case *N* = 9	Control *N* = 45	*p*‐value	OR	95% CI
Age (years)	83.22 ± 9.40	76.76 ± 9.14	0.013	1.351	1.065–1.713
Course of DF (months)	3.11 ± 1.45	2.02 ± 1.08	0.032	1.968	1.058–3.660
Gender			‐	‐	‐
Male	6 (66.7%)	30 (66.7%)			
Female	3 (33.3%)	15 (33.3%)			
Course of DM（years)	27.22 ± 7.12	27.22 ± 4.87	1.000	1.000	0.861–1.161
Ulcer classification (TEXAS)			0.222	0.021	0.000–10.384
3D	‐	17 (37.8%)			
4D	9 (100.0%)	28 (62.2%)			
Education level					
Primary school	5 (55.6%)	39 (86.7%)			
Junior school	3 (33.3%)	6 (13.3%)	0.931	0.000	
College	1 (11.1%)	‐	0.941	0.000	
Course of smoking (years)	22.22 ± 18.56	11.89 ± 17.94	0.080	1.055	0.994–1.120
Smoking			0.071	0.117	0.011–1.201
No	3 (33.3%)	28 (62.2%)			
Yes	6 (66.7%)	17 (37.8%)			
Quantity of smoking (per day)	8.89 ± 7.82	8.00 ± 11.20	0.775	1.012	0.931–1.100
Course of drinking	14.44 ± 14.24	8.22 ± 11.54	0.080	1.084	0.991–1.186
Cerebrovascular disease			0.172	0.345	0.075–1.591
No	4 (44.4%)	31 (68.9%)			
Yes	5 (55.6%)	14 (31.1%)			
Hypertension			0.135	0.276	0.051–1.490
No	2 (22.2%)	23 (51.1%)			
Yes	7 (77.8%)	22 (48.9%)			
Carotid atherosclerosis			0.087	0.274	0.062–1.209
No	4 (44.4%)	34 (75.6%)			
Yes	5 (55.6%)	11 (24.4%)			
Fundus atherosclerosis			0.133	0.002	0.000–6.324
No	1 (11.1%)	40 (88.9%)			
Yes	8 (88.9%)	5 (11.1%)			
Renal insufficiency			0.154	4.980	0.548–45.289
No	8 (88.9%)	29 (64.4%)			
Yes	1 (11.1%)	16 (35.6%)			

Abbreviations: CI, confidence interval; DF, diabetic foot; OR, odd ratios.

The patient's laboratory index test showed that lipoprotein (smLDL), homocysteine (Hcy), superoxide dismutase (SOD), and prealbumin (PA) and other laboratory indexes are risk factors. The higher the value, the greater the risk of death. Each additional unit of laboratory index results for smLDL, Hcy, SOD, and P, The risk of death increased by 29.672, 2.343, 1.028, 1.065 times respectively (Table 2).

**TABLE 2:** Laboratory indicators of patients

Characteristic	Case *N* = 9	Control *N* = 45	*p*‐value	OR	95% CI
smLDL	1.46 ± 0.33	1.10 ± 0.27	0.010	29.672	2.213–397.800
Hcy (umol/L)	17.66 ± 2.22	14.70 ± 1.89	0.005	2.343	1.288–4.262
SOD (U/mL)	240.56 ± 59.98	188.38 ± 34.64	0.010	1.028	1.006–1.050
PA (mg/L)	373.75 ± 72.69	266.45 ± 39.11	0.038	1.065	1.003–1.131
FBG (mmol/L)	12.39 ± 2.30	11.19 ± 2.50	0.155	1.264	0.915–1.746
HbA1c (mmol/L)	18.66 ± 1.99	16.78 ± 3.76	0.124	1.198	0.952–1.508
TC	5.19 ± 0.28	4.92 ± 4.92	0.872	1.012	0.878–1.165
TG	2.92 ± 0.69	1.04 ± 0.18	0.124	105.463	0.277–40103.837
HDL	1.63 ± 0.60	1.38 ± 0.37	0.120	4.238	0.686–26.174
LDL	3.67 ± 0.45	1.87 ± 0.37	0.136	95.390	0.240–37895.020
Alb (g/L)	27.56 ± 3.36	25.38 ± 3.17	0.078	1.285	0.973–1.697
Cr	105.94 ± 77.86	74.82 ± 8.24	0.101	1.078	0.986–1.179
BUN (mmol/L)	15.81 ± 27.98	7.25 ± 0.94	0.288	1.053	0.958–1.157
UA (umol/L)	401.56 ± 62.33	387.84 ± 40.43	0.397	1.007	0.991–1.024
PCT	1.21 ± 0.53	1.25 ± 0.33	0.775	0.756	0.112–5.110
WBC (*10^9^/L)	17.14 ± 3.91	16.20 ± 3.86	0.511	1.061	0.889–1.266
N (*10^9^/L)	10.11 ± 2.08	13.05 ± 3.94	0.054	0.783	0.610–1.004
HGB (g/L)	8.76 ± 0.81	8.56 ± 1.11	0.621	1.173	0.622–2.214
PLT (*10^9^/L)	274.56 ± 71.43	297.53 ± 37.51	0.161	0.988	0.971–1.005
CRP (mg/L)	109.33 ± 35.49	129.71 ± 32.05	0.113	0.978	0.952–1.005

Abbreviations: A1b, albumin; BUN, blood urea nitrogen; Cr, creatinine; CRP, C‐reactive protein; FBG, fasting blood‐glucose; HbA1c, hemoglobin A1C; HDL, high‐density lipoprotein; HGB, hemoglobin; LDL, low‐density lipoprotein; N, neutrophil; PCT, procalcitonin; PLT, platelet; TC, serum total cholesterol; TG, triglyceride; UA, uric acid; WBC, white blood cell.

### In‐hospital Death Forecasting Model

**Fig. 1:**
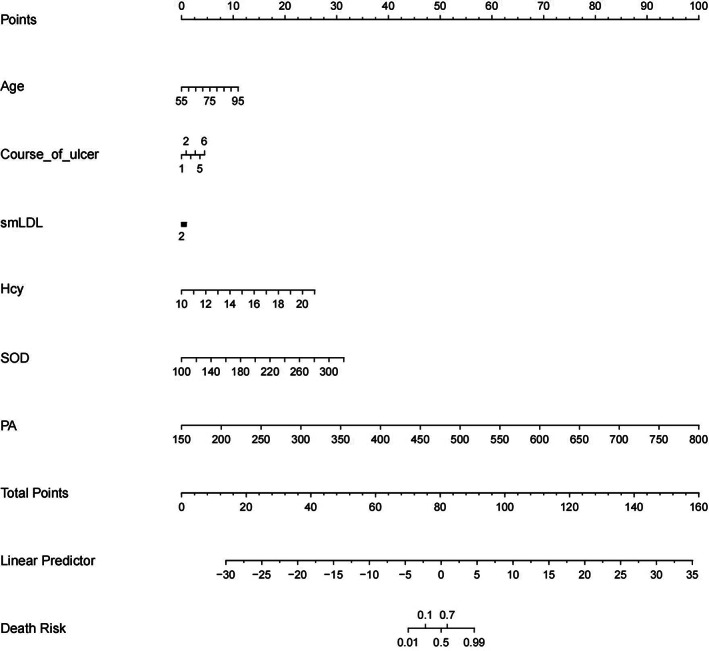
Nomogram of death risk factors.

In this study, a nomogram was employed to assess patients' risk of death by integrating various factors. A nomogram is a visual representation that combines multiple variables to predict a specific outcome. In this case, the findings from the nomogram indicated that the points attributed to pulmonary artery (PA) represented the largest proportion among the death factors. Additionally, the maximum individual points for both the course of ulcer and small dense low‐density lipoprotein (smLDL) were found to be less than 10. It is important to note that when the total points on the nomogram reached 92, the patient's risk of death was as high as 99% (Fig. 1).

**Fig. 2:**
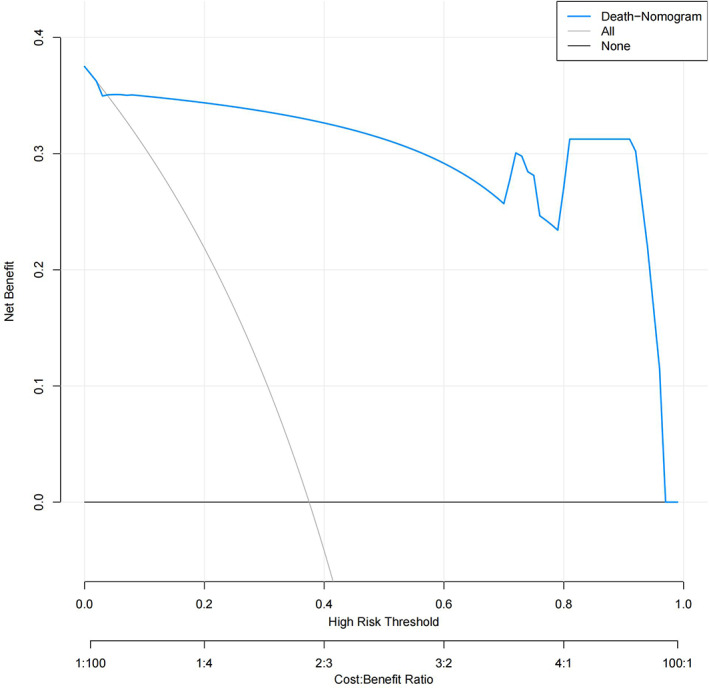
Decision curve analysis (DCA) of total points and patients death risk.

The decision curve analysis is a method used to evaluate the clinical usefulness of a prediction model by weighing the benefits and harms of different treatment strategies. In this case, the decision curve demonstrates that if an individual's threshold probability lies between 2% and 96%, the model in question provides more benefit in predicting death than either the treat‐all or treat‐none tactics. In the specified range of 2% to 96% threshold probability, the prediction model offers a more balanced and accurate method for determining the best course of action for individual patients (Fig. 2).

## Discussion

Death is the worst outcome of DFU that doctors most want to avoid. Although TTT technology has been proved to be an effective means to improve the limb salvage rate of patients with DFU, in‐hospital death is still inevitable. This study identified six risk factors for in‐hospital death in patients with severe DFU treated with TTT, and constructed corresponding predictive models.

### Mortality of DF

Although there are many epidemiological studies on DF, there are relatively few analysis of risk factors for in‐hospital death in DF treatment. A study showed that about 10% of patients with lower limb amputation died during the perioperative period.^15^ In the researcher's clinical practice, nine of 406 patients with chronic ischemic DFU have received TTT treatment died in the hospital so far, and the perioperative mortality rate was 2.22%. Five patients died of lung infection and four patients died of cardiovascular failure among the death cases. Therefore, the blow of TTT might not increase the mortality of DF patients.

DF is the most likely to cause physical disability and even death of patients among all the complications of diabetes.^16^, ^17^, ^18^ DFU is even higher than the mortality and disability rate of most cancers.^5^, ^19^ The literature shows that the mortality rate of DF patients is 43%–50%, and the five‐year survival rate is less than 50%.^20^ In addition, it was estimated that 30% to 50% of DF amputation patients would need to be amputated again within 1 to 3 years.^16^ Major amputations usually resulted in severe functional disability and higher postoperative mortality.^21^ Although there was evidence that with the adoption of active cardiovascular risk management programs, the current mortality rate had decreased.^22^ The overall average survival rates of patients with DF after major amputation in 1, 3, and 5 years are 81%, 69%, and 29%, respectively.^21^ The 245 patients who had undergone toe amputation were followed up for 5 years in Tianjin, China, and the mortality rates were 5.8%, 15.1% and 32.7% respectively.^22^

The etiology of DF ulcers is diverse and the mechanism is complex which is difficult to be understood. Studies have pointed out that ischemia was a key factor leading to the difficulty of healing of DFU wounds.^23^ The periosteum contained a variety of microvessels and growth factors. It was found that a large number of vascular sinuses were formed around the new bone during the fracture healing process when observing the blood supply of the osteogenic area.^24^ These newly formed vascular sinuses were mainly supported by the periosteum, and some have no new angiogenesis. The bone quality was prone to nonunion and so on.^25^ Choi *et al*. found that both the periosteum and the blood vessels in the bone marrow increased, and the amount of blood vessels in the bone marrow was less than that in the periosteum through a rat tibia traction experiment.^26^

### Risk Factors of DF

TTT has been proven to be effective in the treatment of severe chronic ischemic DFU in retrospective studies. Many Chinese physicians had adopted this method to treat chronic ischemic DFU. The effective rate of TTT to DFU was close to 100% in a 7‐year retrospective cohort study.^27^ Improving the peripheral microcirculation of the lower extremities might be the mechanism for this operation to exert its therapeutic effect. TTT periodically transports tibial bone flap to promote the proliferation of blood vessels around the bone masses, forming lower extremity arterial collateral circulation, improving peripheral blood circulation, and promoting DFU of healing.

Age has been proven to be a risk factor for the occurrence of DF.^28^ In this study, it was also proved to be a risk factor for in‐hospital death of elderly patients after receiving TTT treatment. It has been pointed out in the latest national census in China that the old population proportion was increasing, and the influence of increasing age on the prognosis of patients needed to be taken seriously. Studies have shown that the course of DM was a risk factor for the onset of DF,^28^ but it was not a risk factor for in‐hospital death of elderly patients. This study showed that compared with the course of diabetes, the course of DF had a greater impact on in‐hospital death of patients.

The metabolic status of the patient had a significant impact on the death of elderly patients with DF, especially lipid metabolism. Both SOD and smLDL were risk factors for in‐hospital death. Defects in the synthesis and clearance of plasma lipoproteins are the most common cause of abnormal lipid metabolism in diabetes. Diabetic dyslipidemia referred to as the common dyslipidemia in type 2 diabetes characterized by low levels of HDL, hypertriglyceridemia, and postprandial lipemia.^28^ Hypercholesterolemia, LDL levels >130 mg/dL, and hypertriglyceridemia were risk factors for cardiovascular disease in DF, and suggest that dyslipidemia was an important factor in accelerating macrovascular disease in diabetic patients.^29^ But smLDL was more valuable in the prognosis of in‐hospital death of elderly DFU patients receiving TTT.

Contrary to expectations, comorbidities such as vascular disease and renal insufficiency were not risk factors for in‐hospital death in DFU patients. It might be due to the subjects of this study who were elderly people, most of whom had a long medical history, and were often associated with hypertension, cerebral infarction, coronary artery disease, diabetic retinopathy, etc.^2^ Therefore, comorbidities were not a risk factor for in‐hospital death of patients in this population, and it did not mean that comorbidities had no effect on the prognosis of all patients with DFU. Meanwhile, as an important risk factor for atherosclerosis and thrombotic diseases, Hcy was also a risk factor for in‐hospital death in elderly patients with DFU.

The history of drinking and smoking were not the in‐hospital death factors of patients with DFU, but they were the risk factors of ulcer healing. Smoking not only increased the risk of DF, but also delayed the healing of DF wounds and even increased the risk of amputation.^28^ Although the number of alcohol drinkers among diabetic patients was not low, some studies had shown that it was not strongly correlated with the onset of DF.^28^

Despite a variety of treatment methods, the adverse outcome of death has always been a problem that DF patients cannot ignore. Therefore, preoperative evaluation of patients is particularly important. According to the patient ‘s medical disease and related risk factors, medical treatment was first given to improve the patient ‘s indicators, and then surgical treatment was used to treat DFU wounds. Multidisciplinary diagnosis and treatment is still the core of the treatment of DFU.^30^ It is evident from this study that diabetic patients with lower prealbumin levels are more prone to DF complications and death after TTT. Hyperhomocysteinemia is also a factor for death in DF patients. Diabetic patients with elevated plasma homocysteine also had a significantly higher risk of death.^31^ Meanwhile, hyperlipidemia and elevated superoxide dismutase levels are also one of the important factors for death in DF patients, and controlling lipid levels is important for reducing mortality in DF patients.^32^

### Limitations and Strengths

There were a little limitations in this present study. This study was a retrospective study with certain information bias and a relatively small sample size. However, in‐hospital death after TTT treatment of elderly patients with DFU was small probability event which limited the number of cases. A nested case–control study can be conducted to analyze the deaths of such patients in the hospital in the future. Age was also one of the matching criteria at the beginning of the design of this study, but the control group lacked the corresponding elderly patients. Only the oldest patients in the control group who met other matching conditions could be selected. There was a certain bias, which reduced the power of the age. However, age was still proven to be a risk factor for in‐hospital death in elderly patients with DFU undergoing TTT. The nomogram of this study lacked certain external verification. However, the main purpose of this study was to find out the risk factors of in‐hospital death in DFU patients treated with TTT, not to develop a risk prediction model for in‐hospital death. The nomogram could still provide some reference value for the assessment of risk factors of in‐hospital death patients.

## Conclusions

In summary, age, course of DF, smLDL, Hcy, SOD, PA are risk factors for in‐hospital death of patients. These six indicators need to be diligently checked during DFU treatment.

## Author Contributions

Hailin Xu and Junlin Zhou were involved in each of the following points: design; analysis; and funding support. Yusong Yuan and Xiaofang Ding were involved in each of the following points: treatments of patients and manuscript writing. Zhengwei Jing did the analysis and manuscript writing. Hao Lu, Yuanli Wang was involved in in each of the following points: data collection and manuscript revision.

## Funding Information

This study was supported by Capital'sFunds for Health Improvement and Research, No. 2020‐2‐4086; Beijing Health Science and Technology Achievements and Appropriate Technology Promotion Project, No. BHTPP2022015; National Key R&D Program of China, No. 2022YFC2504302; Peking University People's Hospital Scientific Research Development Funds, No. RDL2021‐07; Key Laboratory of Trauma and Neural Regeneration (Peking University), Ministry of Education.

## Conflict of Interest

The author(s) declared no potential conflicts of interest with respect to the research, authorship, and/or publication of this article.

## Ethical Statement

This study was approved by the Ethics Committee of Beijing Long Fu hospital, with the approval number LFYYLL‐2021‐16.
